# Regulation of the calcium-sensing receptor in both stomatal movement and photosynthetic electron transport is crucial for water use efficiency and drought tolerance in *Arabidopsis*

**DOI:** 10.1093/jxb/ert362

**Published:** 2013-11-01

**Authors:** Wen-Hua Wang, Juan Chen, Ting-Wu Liu, Juan Chen, Ai-Dong Han, Martin Simon, Xue-Jun Dong, Jun-Xian He, Hai-Lei Zheng

**Affiliations:** ^1^Key Laboratory for Subtropical Wetland Ecosystem Research of MOE, College of the Environment and Ecology, Xiamen University, Xiamen, Fujian 361005, China; ^2^Key Laboratory for Cell Biology of MOE, School of Life Sciences, Xiamen University, Xiamen, Fujian 361005, China; ^3^Central Grasslands Research Extension Center, North Dakota State University, Streeter, ND 58483, USA; ^4^State Key Laboratory of Agrobiotechnology and School of Life Sciences, The Chinese University of Hong Kong, Hong Kong, PR China

**Keywords:** *Arabidopsis*, calcium-sensing receptor, drought tolerance, stomatal movements, water use efficiency.

## Abstract

Production per amount of water used (water use efficiency, WUE) is closely correlated with drought tolerance. Although stomatal aperture can regulate WUE, the underlying molecular mechanisms are still unclear. Previous reports revealed that stomatal closure was inhibited in the calcium-sensing receptor (CAS) antisense line of A*rabidopsis* (*CASas*). Here it is shown that decreased drought tolerance and WUE of *CASas* was associated with higher stomatal conductance due to improper regulation of stomatal aperture, rather than any change of stomatal density. *CASas* plants also had a lower CO_2_ assimilation rate that was attributed to a lower photosynthetic electron transport rate, leading to higher chlorophyll fluorescence. Gene co-expression combined with analyses of chlorophyll content and transcription levels of photosynthesis-related genes indicate that CAS is involved in the formation of the photosynthetic electron transport system. These data suggest that CAS regulates transpiration and optimizes photosynthesis by playing important roles in stomatal movement and formation of photosynthetic electron transport, thereby regulating WUE and drought tolerance.

## Abstract (precis)

Plant calcium sensing receptor (CAS) optimizes photosynthesis by its effect on the formation of photosynthetic electron transport. CAS also regulates transpiration under water stress. A novel correlation between CAS and plant water use efficiency is revealed

## Introduction

Global climate change has altered the distribution of rainfall, leading to increased frequency and intensity of drought in many areas around the globe, which poses a serious challenge for global agriculture, ecosystems, and plant species distribution (Ciais *et al.*, 2005; Engelbrecht *et al.*, 2007; Passioura, 2007). Plants have evolved to reduce water use when water resources are limited. Under water-stressed conditions, plants reduce photosynthesis by decreasing stomatal conductance and rapidly changing cellular metabolism (Atkin and Macherel, 2009).

Changes in stomatal conductance, which are modulated by stomatal movements and density, directly affect the transpiration rate and CO_2_ uptake, and thereby modulate water use efficiency (WUE) and drought tolerance (Chaerle *et al.*, 2005; Huang *et al.*, 2009). The mechanisms of stomatal development and the consequences of altered stomatal density on drought tolerance and WUE are well understood (Boccalandro *et al.*, 2009; Peterson *et al.*, 2010; Yoo *et al.*, 2010). Furthermore, stomatal movements are the most prominent response to drought, which can be characterized by networks of chemical and molecular signalling pathways including abscisic acid, calcium, and pH signalling, as well as some transcriptional factors (Jia and Zhang, 2008; Cominelli *et al.*, 2010; Zou *et al.*, 2010). Interestingly, recent studies showed that the thylakoid-localized calcium-sensing receptor (CAS) could regulate stomatal movements during calcium signalling transduction (Han *et al.*, 2003; Tang *et al.*, 2007; Nomura *et al.*, 2008). The correlation between CAS and stomatal movements under drought stress is still an open question.

Photosynthesis decreases when plants suffer from drought stress, which results from both stomatal closure that inhibits CO_2_ uptake, and decreased chloroplast activity (Chaves, 1991). Drought stress can also reduce chlorophyll content as well as changes in the ratio of chlorophyll *a* to *b* (Jaleel *et al.*, 2009). In addition, chloroplast thylakoid membranes, containing photosystem I (PSI) and II (PSII), are the crucial site for photosynthetic electron transport. A recent report indicated that chloroplast CAS is crucial for photoacclimation and the expression of LHCSR3, an ancient light-harvesting protein that mediates non-photochemical quenching (NPQ) for effective photoprotection in PSII of *Chlamydomonas reinhardtii* (Petroutsos *et al.*, 2011). Notably, *Piriformospora indica*-colonized Chinese cabbage promotes CAS expression to prevent drought-induced inhibition of photosynthetic efficiency and degradation of chloroplasts (Sun *et al.*, 2010). Interestingly, CAS was further found to be involved in chloroplast-mediated activation of plant immune signalling (Nomura *et al.*, 2012). It is not clear how thylakoid-localized CAS regulates chloroplast activity and photosynthesis, especially under drought stress.

This report investigates the relationships between CAS, WUE, and drought tolerance as well as the potential molecular mechanism(s), using multidisciplinary approaches including a time-course of drought treatment. Infrared gas analysis and chlorophyll fluorescence parameters combined with epidermal bioassay for stomatal aperture and epidermal anatomy were used to indicate the effect of CAS on photosynthesis and transpiration and to explain the underlying cause. In addition, further studies on the potential relationship between CAS and photosynthetic electron transport were performed based on transcriptional as well as web-based co-expression analysis.

## Materials and methods

### Plant materials and growth conditions

Seeds of *Arabidopsis* wild type and *CASas* were sown on half-strength Murashig and Skoog (MS) medium, and 4-day-old seedlings were then transplanted to mixture matrix (peat soil:vermiculite=1:1) and grown under a short-day period of an 8h light and 16h dark cycle with a photon flux rate of 200 μmol m^–2^ s^–1^ provided by a combination of mercury and sodium lamps at 22 ºC, 70% relative humidity. Seeds of the wild type and *CASas* (background Col-0) were obtained from Duke University. To confirm the inhibition of the CAS expression level in *CASas* plants, western blot analysis was performed using purified CAS-specific antibody, and the results showed that CAS expression was severely reduced in *CASas* plants (Supplementary Fig. S1 available at *JXB* online).

### Water deficit stress

Twenty 5-week-old wild-type and *CASas* plants, grown in 500ml pots, were used for water deficit stress treatments. In brief, both wild-type and *CASas* plants were first grown in well-watered pots. Then, water deficit stress was initiated by withholding water until wilting of the lower leaves of the wild-type plant was observed. Both plants were then re-watered and the survival rates were assessed 4 d after re-watering. Fully expanded leaves at different relative soil water contents (SWCs) were excised, and the leaf relative water content (RWC) during this water stress treatment was assessed. Relative SWC and leaf RWC were calculated as described by Yoo *et al.* (2010). Experiments were independently repeated four times. Pots containing these wild-type or *CASas* plants were photographed at different relative SWCs.

### Epidermal bioassay

Detached leaves of *Arabidopsis* seedlings grown under different relative SWCs were blended to separate the epidermis easily from mesophyll tissue, and the resulting epidermal fragments were immediately imaged under a microscope (Motic AE31, Speed Fair Co., Ltd, Hong Kong) to obtain a time-dependent curve of stomatal aperture during the period of water deficit stress treatments based on previously described methods (Pei *et al.*, 1997; Guo *et al.*, 2003). Stomatal apertures were determined as the ratio of width to length using image analysis computer software (SigmaScan Pro5) as previously described (Perera *et al.*, 2008; Wang *et al.*, 2012). To investigate epidermal anatomy, adaxial and abaxial epidermis from the middle portion of the first pair of leaves from 5-week-old plants was peeled gently and then characterized (Yoo *et al.*, 2010). Stomatal density, stomatal index (ratio of stomata to total epidermal cells, including stomata and pavement cells), and pavement cell density were counted from an epidermis area of 0.29mm^2^ under a microscope (×200 magnification). Cell walls were drawn on the image using the brush tool of Photoshop v.7.0 to improve the visualization of both guard cells and pavement cells.

### Whole-plant transpiration rate and thermal imaging

The transpiration rate of 6-week-old whole plants was determined by a gravimetric method (Masle *et al.*, 2005) with minor modification. To avoid evaporation from the soil surface during the measurement of transpiration, 200ml pots containing individual plants were watered at field capacity and covered with a polyethylene wrap. To determine the transpiration rate under a short-term dark to light treatment, individual plants in the pots were first placed in the dark for 2h. The weight of each pot was determined every 10min on a balance for 1h in the dark followed by 2h of light exposure with 200 μmol m^–2^ s^–1^. To measure the diurnal transpiration rate, pots were weighed at the beginning of the photoperiod and 24h later. At the end of the experiment, total leaf area was determined from photographs of excised leaves using Photoshop v.7.0. The transpiration rate and diurnal water loss were finally calculated based on the gravimetric water loss rate divided by the total leaf area from different independent containers. A spline curve was used to fit to the transpiration rate data. Thermal images of plants grown under water stress (WS; relative SWC at 54%) and in well-watered soil (WW; relative SWC at 100%) were obtained using a ThermaCAM™T330 infrared camera (FLIR System), and the leaf temperatures were analysed as described (Miao *et al.*, 2006). The temperature range of thermal images was restricted to between 17.5 ºC and 21.5 ºC. Experiments were repeated five times and four plants were used for each experiment.

### Leaf gas exchange analysis and integrated WUE measurement

Individual 6-week-old *Arabidopsis* plants grown under WW conditions were used for WS treatments by withholding soil water for 8 d when the relative SWC reached WS (~54%) or keeping the relative SWC at ~100% as in WW. Transpiration, stomatal conductance, net CO_2_ assimilation, and intercellular CO_2_ concentration of individual fully expanded leaves from these 8-week-old *Arabidopsis* plants grown under WW or WS conditions were measured by an infrared gas analysis system (Li-6400, Li-Cor, Lincoln, NE, USA). Gas exchange was measured under saturated light at PAR levels of 1000 μmol m^–2^ s^–1^ provided by the red and blue diodes of the gas-exchange system [6400-02B light-emitting diode (LED) light source; LI-COR] in a Li-6400 chamber to measure the potential photosynthesis ability of plants as described in previous reports (Rossel *et al.*, 2007; Yoo *et al.*, 2010; Bermúdez *et al.*, 2012) with actual leaf temperatures between 25 ºC and 27 ºC during measurements. The flow rate of air was set to 500 μmol s^–1^, while the vapour pressure deficit (VPD) was ~1 kPa during measurement. The leaves were left to acclimate to PAR levels of 1000 μmol m^–2^ s^–1^ until CO_2_ assimilation rates were stable. Then all gas exchange parameters were recorded. At the end of the experiment, total leaf area was determined from photographs using Photoshop v.7.0. Net CO_2_ assimilation rates divided by transpiration rates were calculated as the instantaneous WUE. Integrated WUE measurement was carried out for a period of 6 weeks using a gravimetric method (Yoo *et al.*, 2010). Integrated WUE was calculated as the final shoot dry weight divided by total water loss.

### Chlorophyll content and fluorescence measurement

The first and second rosette leaves numbered from the base of 8-week-old plants grown under WW or WS conditions were used to determine the chlorophyll content and fluorescence. Chlorophyll was extracted from leaves with 80% (v/v) aqueous acetone. Absorbance of chlorophyll extracts was measured at 664nm and 647nm with a spectrometer (Varian Cary 50 UV-VIS). The concentration of total chlorophyll or the chlorophyll *a*/*b* ratio was calculated as described by Inskeep and Bloom (1985). The chlorophyll fluorescence ratio was measured using a chlorophyll fluorometer (Fiberoptics PAM-Fluorometer 3050-F) attached to a Portable Gas Exchange Fluorescence System (GSF-3000; Walz, Effeltrich, Germany). The minimum chlorophyll fluorescence at the open PSII centre (*F*_o_) and the maximum chlorophyll fluorescence at closed PSII centres in the dark (*F*_m_) as well as during actinic light illumination (*F*_m_′) were measured after dark adaptation for 30min. The maximum photochemical efficiency of PSII was assayed by calculating the ratio *F*_v_/*F*_m_ [(F_m_–F_o_)/*F*_m_] (Kitajima and Butler, 1975). The photon flux density (PFD) (range from 0 to 1400 μmol m^–2^ s^–1^) provided by LED light source 3040-L (Walz) was gradually increased to measure the light-dependent electron transport rate (ETR) as previously described (Munekage *et al.*, 2002), while steady-state levels of chlorophyll fluorescence (*F*_s_) were measured under an LED light-adapted state of 1000 μmol m^–2^ s^–1^ for 3min. For red chlorophyll fluorescence imaging, the leaf was immediately used to obtain red chlorophyll fluorescence under blue light excitation using a stereo fluorescence microscope (SZX-1LLB2-220, Olympus, Japan).

### Transmission electron microscopy

The leaf chloroplast ultrastructures of 8-week-old plants grown under WW conditions were viewed by transmission electron microscopy (TEM). Hand-cut sections (2mm wide) were obtained from the leaves. The samples were vacuum-inﬁltrated for 5min in primary fixative containing 2.5% (v/v) glutaraldehyde in 0.05M phosphate buffer, pH 7.0 and were then fixed overnight at 4 ºC. After fixation, samples were washed in phosphate buffer and fixed for 1h in 2% (v/v) OsO_4_, followed by dehydrating in an ascending ethanol series. Samples were polymerized for 48h at 60 ºC and the 0.1 μm thick ultrathin sections were prepared, mounted on uncoated copper grids, and then stained with lead citrate for 3min before viewing by TEM (model JEM-2100HC, JEOL, Japan).

### Real-time quantitative PCR analysis

Total RNA was extracted from developing leaves (3 weeks old) using the TRIZOL Reagent (Invitrogen Inc., Carlsbad, CA, USA). For real-time quantitative PCR, first-strand cDNA was synthesized using M-MLV reverse transcriptase (Takara Bio Inc., Japan) with oligo d(T)_18_ primer. The resulting cDNAs were used as templates for subsequent PCRs which were performed on the Rotor-Gene™ 6000 real-time analyzer (Corbett Research, Mortlake, Australia) in standard mode with FastStart Universal SYBR Green (ROX, Roche Ltd, Mannheim, Germany) according to the manufacturer’s protocol. All cycling conditions were as follows: 10min at 94 °C; 45 cycles of 30 s at 94 °C, 30 s at 60 °C, and 30 s at 72 °C; followed by a melting curve program (55 °C to 99 °C, with a 5 s hold at each temperature). The primers were designed according to known sequences of *Arabidopsis STOMAGEN*, *TMM*, *SPCH*, *LHCB3*, *LHCB5*, *PETE1*, and *PSBO2* genes (AT4G12970, AT1G80080, AT5G53210, AT5G54270, AT4G10340, AT1G76100, and AT3G50820, respectively) acquired from the NCBI. The primers used for amplification are listed in Supplementary Table S1 at *JXB* online and the products were checked by melting curve analysis. Amplified products were subjected to sequencing analyses. The mean mRNA expression level was normalized using the ΔΔCt method described by Livak and Schmittgen (2001) and Actin2 as the internal control.

### Statistical analysis

Statistical significance was tested by the one-way or two-way analysis of variance (ANOVA) procedure of SPSS 13.0 (SPSS Inc., Chicago, IL, USA). Post-hoc comparisons were tested using the Tukey or *t*-test at a significance level of *P*<0.05.

## Results

### CAS regulates whole-plant WUE in *Arabidopsis*

**Fig. 1.**
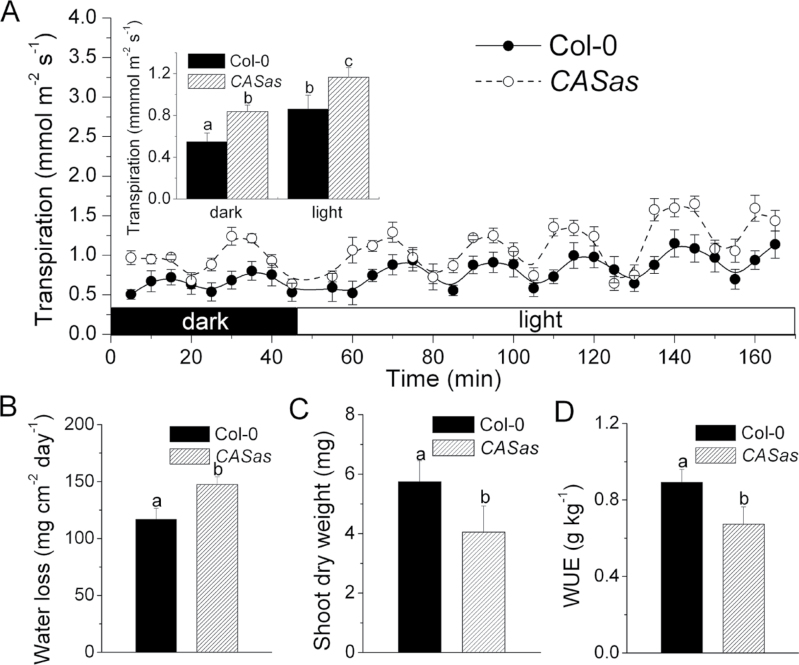
Whole-plant WUE is reduced in *CASas* plants due to excessive transpiration and less biomass production. (A, B) The transpiration rate over 3h of dark to light periods (A) and daily water loss (B) of 6-week-old wild-type (Col-0) and *CASas* plants under well-watered conditions were gravimetrically determined (mean±SE, *n*=4). The inset in (A) shows the average transpiration rate during the dark or light period. (C, D) Shoot dry weight (C) and whole-plant WUE (D) of wild-type and *CASas* plants under well-watered conditions over a period of 6 weeks were calculated from gravimetric measurements (mean±SD, *n*=4). Columns labelled with different letters indicate significant differences at *P*<0.05.

Transpiration rates and biomass produced per plant were measured by gravimetric analyses in both wild-type (Col-0) and *CASas* plants grown in WW conditions to evaluate their WUE. At 6 weeks old, both genotypes showed higher transpiration rates in light than in the dark, while *CASas* plants exhibited higher water loss than wild-type plants over 3h of dark to light periods (Fig. 1A). It is well known that when the periodic variations in transpiration are plotted against time, a sine wave with a period of 10–90min is obtained because of synchronized stomatal activity at the whole-plant level due to the rapid spreading of hydraulic signal (Wallach *et al.*, 2010). Here a period of ~25min was observed (Fig. 1A). Similarly, daily water loss from *CASas* plants was enhanced due to higher transpiration rates (Fig. 1B). The significant increase in transpiration in *CASas* plants was correlated with decreased shoot dry weight (Fig. 1C) or total leaf area over the 6 week period (Supplementary Fig. S2 at *JXB* online). Consequently, *CASas* plants had lower integrated WUE (shoot dry weight/total water loss) than the wild type (Fig. 1D). These results indicate the effect of CAS on WUE regulation in *Arabidopsis*.

### Drought tolerance is weakened by attenuated stomatal closure of *CASas* plants

**Fig. 2.**
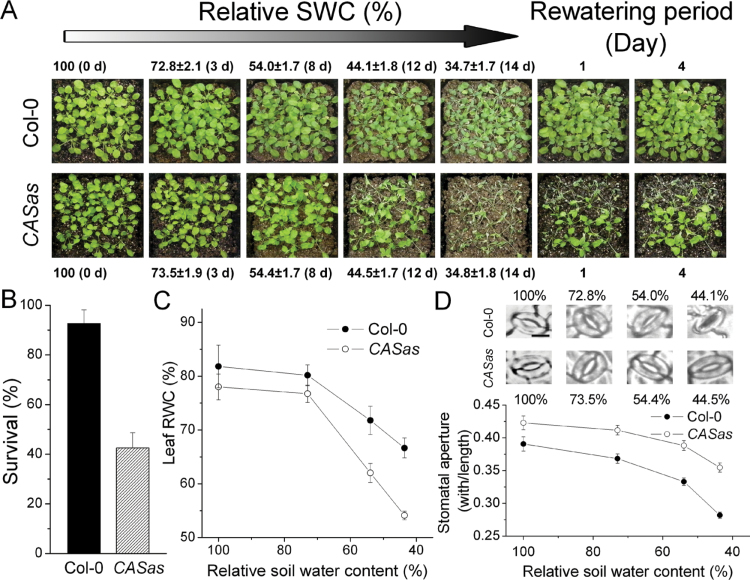
*CASas* plants show reduced survival and maintenance of leaf RWC due to the failure in stomatal closure. Water was withheld from twenty 5-week-old wild-type and *CASas* plants until wilting of the lower leaves of wild-type plants was observed. Both groups of plants were then rewatered and allowed to recover for 4 d. Relative SWC is the soil water relative to the soil water at the beginning of the experiment and is the average of four independent experiments. The photograph in (A) represents results from one replicate. Plant survival (B) was determined 4 d after rewatering (mean±SD, *n*=4). Leaf RWC (mean±SE, *n*=4) (C) and stomatal aperture (mean±SE, *n*=50) (D) from each genotype were immediately determined at different relative SWCs. except at 35%. (This figure is available in colour at *JXB* online.)

To understand the physiological mechanism(s) by which CAS affects transpiration, water was withheld from both wild-type and *CASas* plants, which caused similar water loss from the pots (Supplementary Fig. S3 at *JXB* online). Most of the *CASas* plants wilted at 44% relative SWC, while most of the wild-type plants showed less severe leaf wilting symptoms (Fig. 2A). More than 90% of wild-type and <50% of *CASas* plants survived at a relative SWC of ~35% (Fig. 2B). Consistent with the decreased survival rate, excessive transpiration in *CASas* plants decreased leaf temperature (Supplementary Fig. S4) and leaf RWC (Fig. 2C) compared with the wild type under WS, demonstrating that *CASas* plants were less drought tolerant due to excessive transpiration when grown under low soil moisture conditions.

As shown in Fig. 2D, both wild-type and *CASas* plants exhibited decreased stomatal aperture with the reduction of SWC because of dehydration. However, the stomatal aperture for wild-type plants decreased from 0.391 to 0.282 (by 27.9%) when the SWC dropped from WW to 44%, while for the *CASas* plants it decreased from 0.423 to 0.355 (only by 16.1%), implying that the stomata of *CASas* plants were less sensitive to soil drought than those of wild-type plants, suggesting that CAS is crucial for stomatal closure under WS. Therefore, the high whole- plant transpiration and the low survival rate under WS in *CASas* may be attributed to inadequate regulation of stomatal closure when exposed to drought. These results suggested that CAS is involved in the process of stomatal closure to reduce transpiration.

### Leaf surface characteristics of wild-type and *CASas* plants

**Fig. 3.**
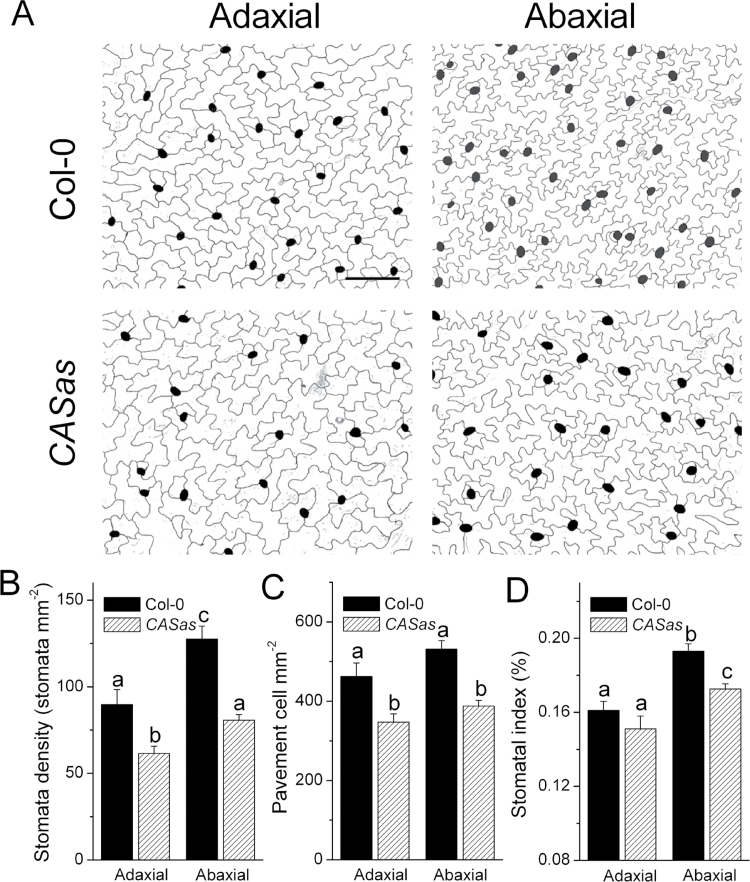
Epidermal anatomy of wild-type and *CASas* leaves. (A) Representative images of leaf adaxial and abaxial epidermal layers from the middle portion of 5-week-old wild-type and *CASas* leaves. Pavement cells and stomata are illustrated in white and black, respectively. Bars=50 μm. (B–D) Stomatal density (the number of stomata per area) (B), pavement cell density (the number of pavement cells per area), (C) and stomatal index (the number of stomata per total epidermal cells) (D) were analysed in the leaf adaxial and abaxial epidermal layers, using images from (A). Data are the mean of seven individual plants (mean±SE, *n*=7). Columns labelled with different letters indicate significant differences at *P*<0.05.

To investigate whether stomatal density is also the basis for the excessive water loss in *CASas* plants, the adaxial and abaxial epidermal anatomy was analysed. Unexpectedly, *CASas* plants had lower stomatal density on both epidermes compared with wild-type plants (Fig. 3A, B). In addition, leaves of *CASas* plants had larger pavement cells (Fig. 3A), which resulted in a lower pavement cell density (Fig. 3C) than that of wild-type plants. Larger pavement cells, which may be attributed to unrepressed endoreduplication (Breuer *et al.*, 2009), probably caused the reduction of stomatal density in *CASas*. However, the abaxial stomatal index (number of stomata divided by total number of epidermal cells) was significantly reduced in *CASas* plants (Fig. 3D), suggesting that differences in stomatal density could also result from inconsistent stomatal differentiation among genotypes. Here, it is shown that the transcription level of *STOMAGEN*, which promotes stomatal differentiation through SPEECHLESS (SPCH) and TOO MANY MOUTHS (TMM) (Sugano *et al.*, 2010), was significantly inhibited in developing leaves of *CASas* plants, while *SPCH* and *TMM* were not disrupted (Supplementary Fig. S5 at *JXB* online). Hence, lower STOMAGEN expression led to lower stomatal density and should inhibit the excessive transpiration in *CASas* plants. However, a remarkable transpiration rate in *CASas* plants was observed (Figs 1A, B, 2A). This may be directly caused by the failure in stomatal regulation (Fig. 2D) but not by the stomatal density of *CASas* plants.

### CAS is related to optimal photosynthesis and effective WUE under WS

**Fig. 4.**
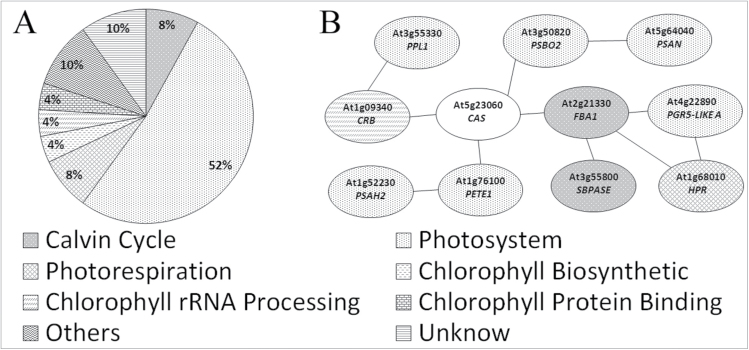
Co-expression analysis of *CAS* and photosystem genes. (A) *CAS* co-expression was analysed based on the current database in the GeneCAT tool (http://genecat.mpg.de/). The returned top 50 genes were statistically summarized in eight groups. (B) Co-expression genes directly connected to *CAS* were found by using ATTED-II (http://atted.jp/).

Gene co-expression provides powerful information to identify relationships between genes, which could reveal the potential function of CAS in plant physiological processes. Using the Co-expression Analysis tool in GeneCAT (http://genecat.mpg.de/) as described (Mutwil *et al.*, 2008), the expression profile of the *Arabidopsis CAS* gene (accession no. At5g23060) was compared with that of every related gene from the current database. The top 50 genes that were co-expressed with *CAS* were summarized in Fig. 4A, and Supplementary Table S2 at *JXB* online. The results showed that most of the genes co-expressed with *CAS* were correlated with photosystem (52%). In addition, genes of the chlorophyll biosynthetic process seemed to be expressed in line with *CAS*. Interestingly, nine different chlorophyll *a*/*b*-binding protein genes, involved in the formation of the photosynthesis light-harvesting complex (LHC), were co-expressed with *CAS* (Supplementary Table S3). This is consistent with a recent report showing that *CAS* knockdown *Chlamydomonas* lines were unable fully to induce LHCSR3, a crucial protein for NPQ (Peers *et al.*, 2009; Petroutsos *et al.*, 2011). Further analysis using ATTED-II (http://atted.jp/) showed that two important photosystem-related genes PLASTOCYANIN 1 (*PETE1*) and PHOTOSYSTEM II SUBUNIT O-2 (*PSBO2*), as well as a Calvin cycle-related gene FRUCTOSE-BISPHOSPHATE ALDOLASE 1 (*FBA1*) interacted with *CAS* (Fig. 4B). STN8 protein kinase, involved in CAS phosphorylation (Vainonen *et al.*, 2008), was also co-expressed with the *CAS* gene (Supplementary Fig. S6). These results together indicated the underlying functional relationship between CAS and plant photosynthesis.

**Fig. 5.**
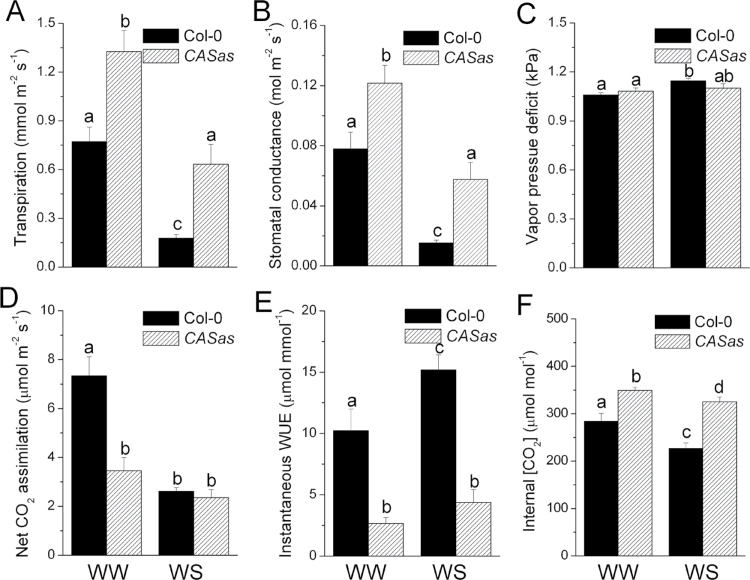
Gas exchange of wild-type and *CASas* plants under well-watered and water stress conditions. Leaf transpiration (A), stomatal conductance (B), VPD (C), net CO_2_ assimilation (D), instantaneous WUE (E), and internal CO_2_ concentration (F) were determined on individual leaves of 8-week-old wild-type and *CASas* plants grown under well-watered (WW) and water stress (WS) conditions at PAR levels of 1000 μmol m^–2^ s^–1^ using a Li-6400 gas exchange system (mean±SE, *n*=5). Columns labelled with different letters indicate significant differences at *P*<0.05.

The instantaneous photosynthesis and WUE of both wild-type and *CASas* plants were determined by an infrared gas analyser to reveal the correlation between CAS, photosynthesis, and WUE, as well as the drought tolerance. After WW and WS treatment for 8 d (Supplementary Fig. S7A at *JXB* online), leaf transpiration and stomatal conductance of *CASas* plant were higher than those of wild-type plants (Fig. 5A, B), which was consistent with the results in Fig. 1A and B. However, these results were not due to different leaf-to-air VPD (Fig. 5C), indicating that the higher transpiration of *CASas* plant was due to the oversized stomatal aperture. In contrast, significant reduction of the net CO_2_ assimilation rate in *CASas* plants was observed (Fig. 5D). Consequently, *CASas* plants had lower instantaneous WUE (CO_2_ assimilation/transpiration) (Fig. 5E). In addition, a higher internal CO_2_ concentration in leaves of *CASas* plant was observed due to the larger stomatal aperture (Fig. 5F), suggesting that decreased photosynthesis in *CASas* plant was not due to stomatal limitation. Interestingly, further analysis showed that the wild-type plants had relatively lower (24.6%) transpiration and lower (27.4%) stomatal conductance but higher (9.4%) leaf RWC than *CASas* plants under WS conditions (Fig. 5A, B; Supplementary Fig. S7B), suggesting the inhibition of stomatal closure under the WS condition in *CASas* plants. However, wild-type plants reduced transpiration to a much greater extent than CO_2_ assimilation, leading to an improved WUE when experiencing WS, while no significant change of WUE was observed in *CASas* plants because of less responsive stomatal aperture (Fig. 5E). Taken together, these results indicated that CAS is crucial for reducing transpiration to a greater extent than photosynthesis in response to WS. This function ensures lower water costs for CO_2_ assimilation under water deficit conditions, leading to the improved WUE and drought tolerance of plants.

### Defective photosynthetic electron transport in *CASas* plants

**Fig. 6.**
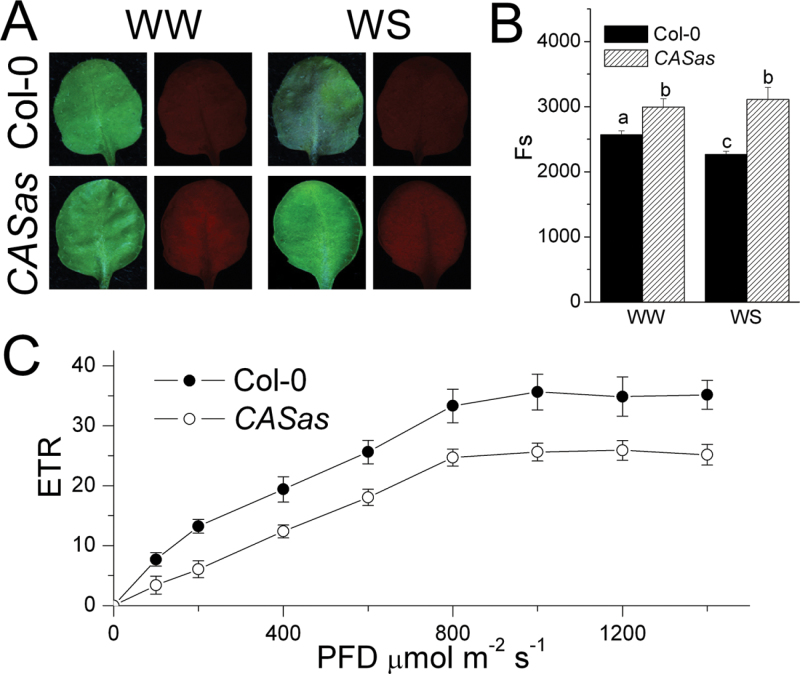
Defective photosynthetic electron transport in *CASas* plants. (A) Leaf chlorophyll autofluorescence imaging of wild-type and *CASas* plants grown under well-watered (WW) and water stress (WS) conditions under blue light excitation using a stereo fluorescence microscope. Experiments were repeated four times. (B) The steady-state levels of chlorophyll fluorescence (*F*_s_) from wild-type and *CASas* leaves grown under WW and WS conditions were measured under an LED-light-adapted state of 1000 μmol m^–2^ s^–1^ for 3min. Data are the means and SE of six biological replicates. (C) A gradually increased photon flux density was applied to measure the light-dependent electron transport rate (ETR) under WW conditions (mean±SE, *n*=6). Columns labelled with different letters indicate significant differences at *P*<0.05. (This figure is available in colour at *JXB* online.)

The co-expression analysis showed that CAS was well correlated with some elements of photosystem electron transport (Fig. 4). Next, the focus was on the function of CAS in photosynthetic electron transport to address its non-stomatal effects on photosynthesis. Here, it was found that leaves of *CASas* plants exhibited higher red autofluorescence emission (Fig. 6A). Consistently, steady-state levels of chlorophyll fluorescence (*F*_s_), a parameter reflecting the autofluorescence emission, were higher in *CASas* and decreased in wild-type plants under WS (Fig. 6B). The higher chlorophyll fluorescence of the *CASas* plant leaf suggested a defect in photosynthetic electron transport and lower utilization of light energy that might result from a less efficient connection between the major LHCII complex and PSII reaction centre (Munekage *et al.*, 2002; Shikanai *et al.*, 2003). The additional data on NPQ showed that the changes in NPQ were almost inversely matched by the changes in *F*_s_ (Supplementary Fig. S8 at *JXB* online). This result also suggested the defect in photosynthetic electron transport in *CASas* plants. The photosynthetic electron transport deficiency in *CASas* plants was further confirmed by determining the PSII ETR. Figure 6C shows that the maximal ETR in *CASas* was ~70% of that in the wild type. In *CASas* under WW conditions, the ETR was decreased even at a low light intensity (100 μmol m^–2^ s^–1^), which explains the reduced biomass production or WUE (Fig. 1) and higher chlorophyll fluorescence. These results demonstrated that *CASas* can decrease the ETR and inhibit photosynthesis.

### CAS participates in the formation of the photosynthetic electron transport system

**Fig. 7.**
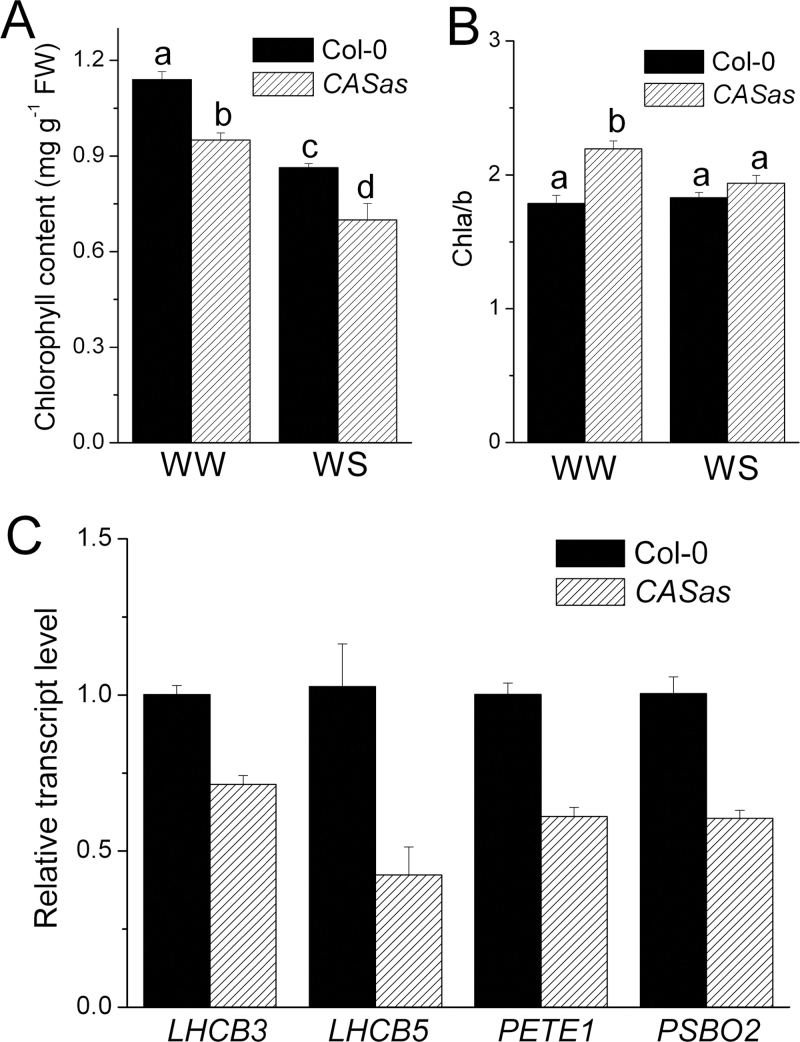
The chlorophyll content and transcription level of photosystem-related genes were disturbed in *CASas* plants. (A, B) Chlorophyll content (A) and ratio of chlorophyll *a* to *b* (B) from wild-type and *CASas* leaves grown under WW and WS conditions were determined (mean±SE, *n*=4). Columns labelled with different letters indicate significant differences at *P*<0.05. (C) Transcription level of four photosystem-related genes: *LHCB3*, *LHCB5*, *PETE1*, and *PSBO2* (mean±SE, *n*=4).

The cause of photosynthetic electron transport deficiency in *CASas* plants was then investigated. A similar organization of stroma membranes and interconnecting grana stacks was observed in chloroplasts from both wild-type and *CASas* plants (Supplementary Fig. S9 at *JXB* online), suggesting that the reduced ETR in *CASas* plants did not result from the lack of integrity in chloroplast structure. However, further analysis showed that the amount of chlorophyll per fresh weight was decreased in *CASas*, especially in WS conditions (Fig. 7A). In contrast, the ratio of chlorophyll *a* to *b* was increased in *CASas* plants (Fig. 7B), reflecting the lower abundance of LHCII that contains chlorophyll *b*. Investigating the expression of the relevant genes in developing leaves may suggest the cause of the changed physiology that is observed in mature leaves. Consistent with previous results, reduced transcript levels of *LHCB3* and *LHCB5* in LHCII as well as of *PETE1* and *PSBO2* (Fig. 7C) were found in developing leaves of *CASas* plants, suggesting that the defect in CAS could down-regulate the gene expression associated with the photosynthetic electron transport system. These data supported the results in co-expression analysis (Fig. 4). These impaired components in the photosynthetic electron transport system could reduce ETR induction in *CASas* plants.

## Discussion

**Fig. 8.**
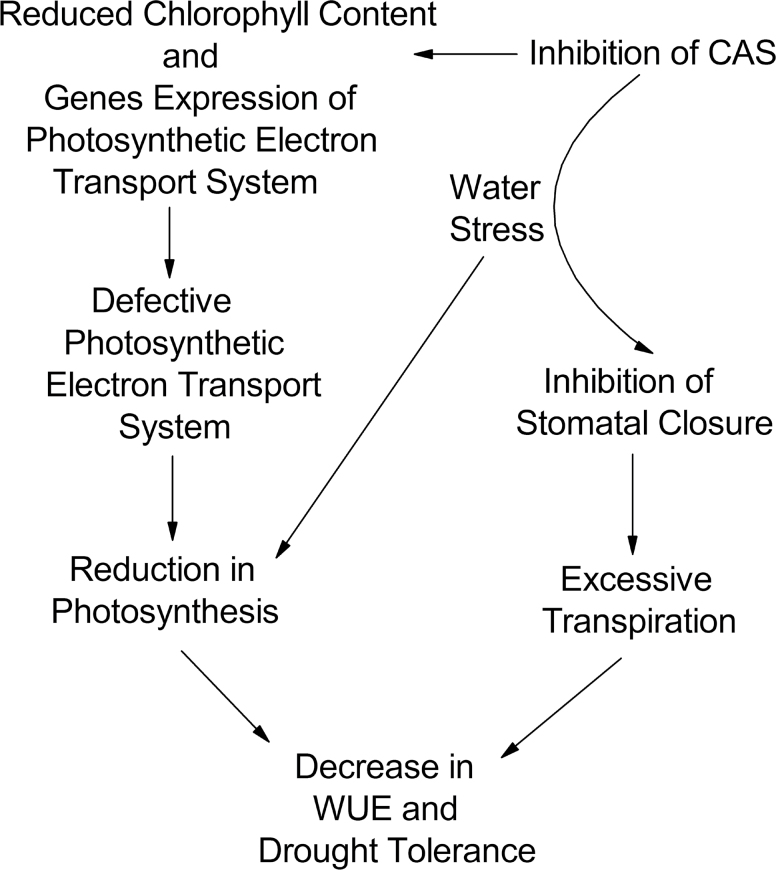
Conceptual model of the effects of *CAS* on plant WUE regulation and drought tolerance. The inhibition of the *CAS* gene decreases chlorophyll content and transcription of photosynthetic electron transport-related genes, leading to defective photosynthetic electron transport which aggravates the reduction of plant photosynthesis in water stress. On the other hand, the inhibition of the *CAS* gene disturbs stomatal closure under water stress and results in excessive transpiration. Finally, the WUE is decreased and the plant dies because of its lower tolerance to drought stress.

In this study, the focus was on the function of CAS in regulating gas exchange and stomatal movements to evaluate the effect of CAS on plant WUE and drought tolerance (Fig. 8). As indicated in the model, photosynthesis is decreased under WS. Moreover, the inhibition of CAS causes defective photosynthetic electron transport (Fig. 6), and a decrease of chlorophyll content and expression of photosynthetic electron transport-related genes (Fig. 7). Furthermore, *CASas* exhibits attenuated stomatal closure in response to WS (Fig. 2D), leading to higher stomatal conductance and excessive transpiration (Fig. 5A, B), thus decreasing WUE (Fig. 5E) and causing lower tolerance to WS (Fig. 2B). This model allowed a systematic understanding of the contribution of CAS to plant drought tolerance.

### Two strategies for regulating WUE through CAS under diverse soil water status

Limiting water loss by reducing stomatal conductance also suppresses the photosynthesis due to decreased CO_2_ uptake and chloroplast activity (Udayakumar *et al.*, 1998; Chaves *et al.*, 2003). The present data revealed that CAS acts as an important mediator to face this dilemma under WS. Given an unlimited water supply, plants maintained a high rate of photosynthesis accompanied by high stomatal conductance (Fig. 5B, D). The existence of CAS in developing chloroplasts guaranteed chlorophyll synthesis and the expression of genes associated with the photosynthetic electron transport system (Fig. 7), leading to better photosynthetic electron transport (Fig. 6) and efficient photosynthesis. In contrast, stomatal opening due to depressed expression of CAS caused high water loss and decreased WUE in *CASas* plants.

In drought environments, the plants maintain the leaf water status (Fig. 2C) and limit stomatal conductance (Fig. 5B) to prevent the risk of more water depletion and their death. The reduced stomatal conductance was triggered by CAS-involved stomatal movement under decreasing SWC (Fig. 2D). Although photosynthesis was inhibited under WS due to reduced stomatal conductance and ETR, the limitation of water loss by the presence of CAS became more dominant, thus improving WUE under WS (Fig. 5). However, the water loss from *CASas* plants was less limited because of the failure of stomatal closure, leading to a minimal improvement in WUE under WS. In addition, down-regulation of *STOMAGEN* was found in *CASas* plants (Supplementary Fig. S5 at *JXB* online), and this might decrease stomatal density and prevent the excessive transpiration in *CASas* plants, suggesting the cooperation between CAS and STOMAGEN in regulating the whole stomatal conductance to maintain the leaf water status.

Furthermore, higher chlorophyll fluorescence as well as lower NPQ, especially under WS, were observed in *CASas* plants (Fig. 6), suggesting impaired photoprotection in the absence of CAS. Leaf temperature dissipation is mainly driven by water loss; however, the excess energy absorbed by the antenna is also harmlessly dissipated as heat to prevent damage observed as NPQ, which is linearly related to heat dissipation (Maxwell and Johnson, 2000; Andersson *et al.*, 2001). It is known that NPQ is caused mainly by the thermal dissipation of excitation energy from PSII, which is induced by electron transport (Munekage *et al.*, 2002). This result also suggested that *CASas* plants were unable to dissipate the excess excitation energy by NPQ under WS. Therefore, the impaired NPQ might also aggravate the leaf damage because of excessive light energy without efficient energy dissipation. Excess energy is potentially harmful and can enhance the production of reactive oxygen species (Mittler, 2002), resulting in photoinhibition of PSII reaction centres (Yuan *et al.*, 2005), damage of ATP synthase (Lawlor and Tezara, 2009), and severe inhibition of photosynthesis and growth (Lawlor and Cornic, 2002; Chaves *et al.*, 2003). *CASas* plants were consequently unable to survive under WS. Taken together, CAS may play important roles in maintaining the plant water status and regulating plant WUE. The effects of CAS on the ETR and photoprotection ability were also advantages that improve the drought tolerance in *Arabidopsis*.

### CAS is possibly involved in transcription of photosynthesis-related genes

*CASas* plants clearly showed impaired photosynthesis. It was reported that the transcription level of *CAS* was significantly up-regulated and phosphorylated under light (Piippo *et al.*, 2006; Vainonen *et al.*, 2008). Furthermore, some chlorophyll biosynthesis- and photosystem-related genes including most LHCs were co-expressed with the *CAS* gene (Fig. 4), while a reduced transcript level of *LHCB3*, *LHCB5*, *PETE1*, and *PSBO2* as well as low chlorophyll content were also detected in developing *CASas* leaves (Fig. 7). Repression of CAS might impair the long-term response by interfering with a signalling pathway that links changes in photosynthetic efficiency to the expression level of light-related photosynthetic genes. The C-terminus of CAS has a rhodanese-like protein domain frequently associated with other domain structures involved in signal transduction (Bordo and Bork, 2002; Han *et al.*, 2003). The C-terminus was also predicted to contain a motif for interaction with 14-3-3 proteins and FHA domains (Vainonen *et al.*, 2008), which are involved in signal transduction and stress responses as well as protein import into chloroplasts (Fulgosi *et al.*, 2002). The possible roles of CAS are currently under investigation.

In conclusion, inhibition of CAS down-regulates transcription of photosynthetic electron transport-related genes, disturbs the formation of the photosynthetic electron transport system, and consequently reduces the ETR, chlorophyll content, and photosynthesis of *CASas* plants, especially under WS. In addition, the absence of CAS also causes the failure of stomatal closure in response to WS, leading to poor stomatal control and excessive plant transpiration. Finally, those changes decrease whole-plant WUE and drought tolerance under WS conditions.

## Supplementary data

Supplementary data are available at *JXB* online.

Figure S1. Verifying the *CASas* line using western blot.

Figure S2. Total leaf area was reduced in *CASas* plants.

Figure S3. Quantification of water loss from plots during each period of drought treatment.

Figure S4. Drought-induced leaf thermal radiation was disrupted in *CASas* plants.

Figure S5. Transcription level of three stomata differentiation genes.

Figure. S6. Correlation of development expression pattern between *CAS* and *STN8* genes.

Figure S7. Relative SWC and leaf RWC at the time of gas exchange measurement for plants under WW and WS conditions.

Figure S8. NPQ measurements of plants in response to drought treatment.

Figure S9. Thylakoid ultrastructure of mesophyll chloroplasts from plant leaves grown under WW conditions.

Table S1. Primer sequences used for RT-PCR analysis.

Table S2. Detailed accession numbers of the top 50 genes in Fig. 4A.

Table S3. Light harvesting complex genes that are co-expressed with *CAS* from the top 150 genes analysed using the GeneCAT tool (http://genecat.mpg.de/).

Supplementary Data
