# Supplementary material for: Regulation of the calcium-sensing receptor in both stomatal movement and photosynthetic electron transport is crucial for water use efficiency and drought tolerance in Arabidopsis
Source: J Exp Bot. 2013 Nov 1;65(1):223–34. doi: 10.1093/jxb/ert362 (PMC3883291; doi:10.1093/jxb/ert362)
Supplement: Supplementary Data [file supp_65_1_223__index.html]

Regulation of the calcium-sensing receptor in both stomatal movement and photosynthetic electron transport is crucial for water use efficiency and drought tolerance in Arabidopsis — Regulation of the calcium-sensing receptor in both stomatal movement and photosynthetic electron transport is crucial for water use efficiency and drought tolerance in Arabidopsis — Supplementary Data 

# Regulation of the calcium-sensing receptor in both stomatal movement and photosynthetic electron transport is crucial for water use efficiency and drought tolerance in *Arabidopsis*

## Supplementary Data

Data files

**Files in this Data Supplement:**

- Supplementary Data - Supplementary Data
