# Supplementary material for: Regulation of the calcium-sensing receptor in both stomatal movement and photosynthetic electron transport is crucial for water use efficiency and drought tolerance in Arabidopsis
Source: J Exp Bot. 2013 Nov 1;65(1):223–34. doi: 10.1093/jxb/ert362 (PMC3883291; doi:10.1093/jxb/ert362)
Supplement: Supplementary Data [file supp_ert362_jexbot093203_file001.pdf]

Journal of Experimental Botany

Title: Regulation of calcium sensing receptor in both stomatal movement and photosynthetic electron transport is crucial for water use efficiency and drought tolerance in Arabidopsis

Authors: Wen-Hua Wang [1], Juan Chen [1], Ting-Wu Liu [1], Juan Chen [1], Ai-Dong Han [2], Martin Simon [1], Xue-Jun Dong [3], Jun-Xian He [4] and Hai-Lei Zheng [1*]

[1] Key Laboratory for Subtropical Wetland Ecosystem Research of MOE, College of the Environment and Ecology, Xiamen University, Xiamen, Fujian 361005, China

[2] Key laboratory for Cell Biology of MOE, School of Life Sciences, Xiamen University, Xiamen, Fujian 361005, China

[3] Central Grasslands Research Extension Center, North Dakota State University, Streeter, ND 58483, USA

[4] State Key Laboratory of Agobiotechnology and School of Life Sciences, The Chinese University of Hong Kong, Hong Kong, P.R. China

[*] To whom correspondence should be addressed. E-mail zhenghl@xmu.edu.cn; Tel: +86 592-218-1005; Fax: +86 592-218-1015.

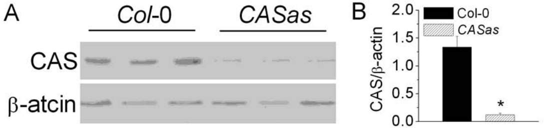

Fig. S1. Verifying the *CASas* line using western blot. (A) The CAS protein expression in WT and *CASas* by western blot. β-Actin was used as an internal control. (B) Densitometric quantification of CAS protein levels for both WT and *CASas* were normalized to β-actin levels. Means and SE were calculated from three replicates;$^{*}P < 0.001$.

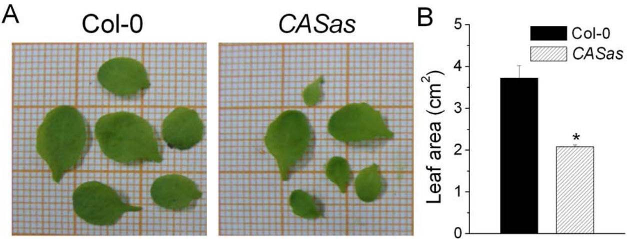

Fig. S2. Total leaves area was reduced in *CASas* plants. Photos of representative leaves morphology (A) and total leaves area (B) of 6-week-old wild type and *CASas* plants used for transpiration experiment in Fig. 1A (mean ± SE, $n = 4$; $*P < 0.001$).

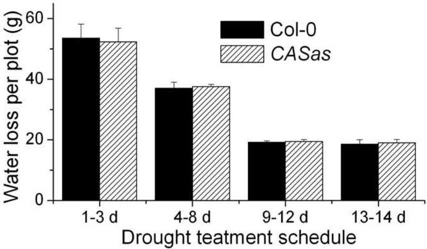

Fig. S3. Quantification of water loss from plots during the each period of drought treatment. Water loss for pot containing WT or *CASas* plants was determined by directly weighing pots at the beginning and at the end of each period of drought treatment (mean ± SE, $n = 4$).

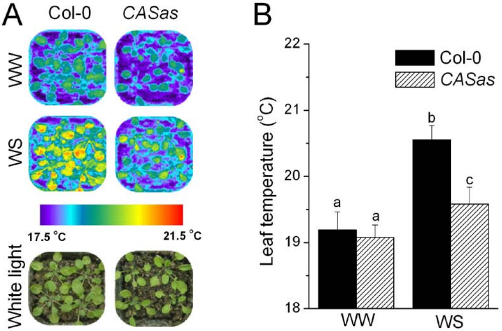

Fig. S4. Drought-induced leaf thermal radiation was disrupted in *CASas* plants. (A) Typical thermal images of rosette leaves in 6-week-old wild-type and *CASas* leaves grown under WW and WS condition represented one from the five independent experiments. Temperature range was restricted between 17.5°C and 21.5°C. (B) Temperature of the leaf surface from (A) quantified by infrared thermal imaging. Data are means ± SD (n = 20 plants for each condition in five independent experiments). Columns labeled different letters indicate significant differences at $P < 0.05$.

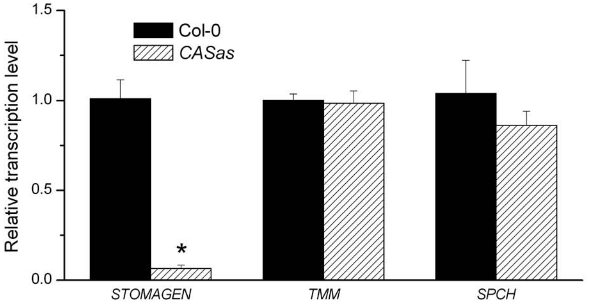

Fig. S5. Transcription level of three stomata differentiation genes.
Three stomata differentiation genes including *STOMAGEN*, *TMM* and *SPCH* were evaluated the transcription levels in developing leaves from 3-week-old wild type and *CASas* plants. Data are means and SE of three biological replicates; $^*P < 0.001$.

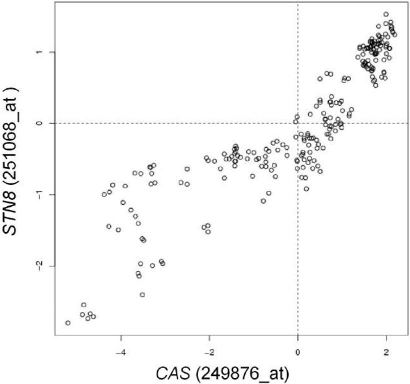

Fig. S6. Correlation of development expression pattern between *CAS* and *STN8* genes. Both axes are relative gene expression values in base-2 logarithm against the averaged expression levels of each gene with the correlation constant evaluated at 0.71 using ATTED-II (http://atted.jp/). Probes for *CAS* and *STN8* are 249876_at and 251068_at, respectively.

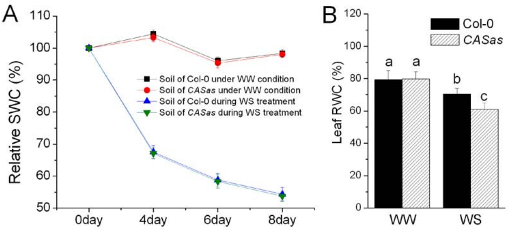

Fig. S7. Relative SWC and leaf RWC at the time of gas exchange measurement for plants under WW and WS condition. (A) Relative SWC of wild type or *CASas* plant during 8-day-treatment of WW or WS (mean ± SD, n = 5). (B) Leaf RWC of wild type or *CASas* plant for gas exchange measurement is determined at the 8th day in (A) when relative SWC reaches to 54% (mean ± SD, n = 5). Columns labeled with different letters indicate significant differences at $P < 0.05$.

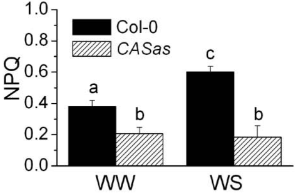

Fig. S8. NPQ measurements of plants in response to drought treatment.
Non-photochemical quenching (NPQ) from 8-week-old wild-type and *CASas* leaves grown under WW and WS condition were measured under LED-light-adapted state of 1000 μmol m^{-2} s^{-1} for 3 min. Data are means and SE of six biological replicates. Columns labeled different letters indicate significant differences at $P < 0.05$.

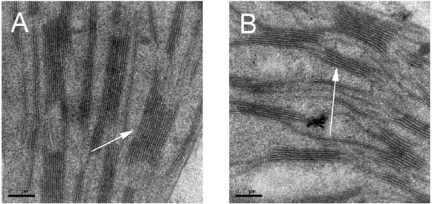

Fig. S9. Thylakoid ultra-structure of mesophyll chloroplasts from plants leaves grown under WW condition. (A) Thylakoid ultra-structure of wild type. (B) Thylakoid ultra-structure of *CASas* . Arrows indicate grana. Scale bars = 200 nm

Table S1. Primer sequences used for RT-PCR analysis

Gene Name		Primer Sequences
STOMAGEN	F	5’- TTATGTAGTTCAAGCCTCAAGACCTCG-3’
	R	5’- CACATCTATAATGATAAGCACTGTTGA-3’
TMM	F	5’- CGGGTCCTTCACCTAGAGGGCAATA-3’
	R	5’- TCTCCTCATCCTCCACACTGTGTCT-3’
SPCH	F	5’- CGATCATAGGAGGAGTTGTGGAGTA-3’
	R	5’- CTTAGAACAGGCGGTGAAGGACGAG-3’
LHCB3	F	5’- GTTGGGTCTCGCTGATGATCCAGTTAC-3’
	R	5’- ACAGGGTTGTCAAGATGGTCAAGGAGA-3’
LHCB5	F	5’- CGGATTGGATTTCGAGGACAAGCTACA-3’
	R	5’- GATAAAGAAACCGAGCATCGCAAACAT-3’
PETE1	F	5’- GGTGTTCGACGAAGACGAGATCCCTAG-3’
	R	5’- TGACGGTGAGTTTCCCAACCATACCAG-3’
PSBO2	F	5’- GCAAGACTTACATGGAGGTAAAGGGTA-3’
	R	5’- GGTGAAGGAAGTGGGCTCGAAGCAGAA-3’
ACTIN2	F	5’-AACTCTCTGGGTTTTTACTTACGTCTGCG-3’
	R	5’-AGGGAACAAAAGGAATAAAGAGGCATCAA-3’

These primer pairs were used as gene specific primers in RT-PCR analysis. F is the forward and R the reverse primer.

Table S2. Detailed accession numbers of the top 50 genes in Fig. 4a.

Group	Gene Accession Number
Calvin Cycle	at1g42970, at1g32060, at3g55800, at1g12900
Photosystem	at1g52230, at1g67740, at3g50820, at4g01050, at3g16140, at1g76100, at4g21280, at4g02770, at4g28750, at5g66190, at1g15820, at5g54270, at4g05180, at4g12800, at2g20260, at4g09650, at1g31330, at3g54890, at1g08380, at3g21055, at3g56940, at1g03130, at3g16250, at5g51010, at5g64040
Photorespiration	at5g36790, at5g36700, at2g35370, at5g04140
Chlorophyll Biosynthetic	at3g59400, at1g03630
Chlorophyll rRNA Processing	at3g63140, at1g09340
Chlorophyll Protein Binding	at1g75690, at1g55480
Others	at5g38520, at5g19940, at1g32470, at1g14345, at4g09010
Unknown	at1g74730, at1g21500, at2g35260, at1g52220, at2g42220

Table S3. Light Harvesting Complex genes that are co-expressed with *CAS* from the top 150 genes analyzed using GeneCAT tool (http://genecat.mpg.de/).

Gene Accession Number	Gene Name
at5g01530	Chlorophyll A-B binding protein CP29 (LHCB4)
at4g10340	Chlorophyll A-B binding protein CP26, chloroplast / light-harvesting complex II protein 5 / LHCIIc (LHCB5)
at1g61520	Chlorophyll A-B binding protein / LHCI type III (LHCA3,1)
at1g19150	Chlorophyll A-B binding protein, putative / LHCI type II
at3g27690	Chlorophyll A-B binding protein (LHCB2:4)
at5g54270	Chlorophyll A-B binding protein / LHCII type III (LHCB3)
at1g15820	Chlorophyll A-B binding protein, chloroplast (LHCB6)
at3g61470	Chlorophyll A-B binding protein (LHCA2)
at3g54890	Chlorophyll A-B binding protein / LHCI type I (CAB)
